# Supplementary material for: Rewriting nuclear epigenetic scripts in mitochondrial diseases as a strategy for heteroplasmy control
Source: EMBO Mol Med. 2025 Aug 11;17(9):2354–83. doi: 10.1038/s44321-025-00285-5 (PMC12423320; doi:10.1038/s44321-025-00285-5)
Supplement: Supplementary file 4 — Source data Fig. 2 [file 44321_2025_285_MOESM4_ESM.zip › Fig 2/2A/read me 2A.docx]

DNA methylation values provided form the 35CpG sites that had at least in one sample >0% methylation.

When there are more than one CpG sites related to one gene, we labeled as 1 or 2.

In the excel file you will see the input chart (before clustering) and the output chart (after clustering).
