# Supplementary material for: Rewriting nuclear epigenetic scripts in mitochondrial diseases as a strategy for heteroplasmy control
Source: EMBO Mol Med. 2025 Aug 11;17(9):2354–83. doi: 10.1038/s44321-025-00285-5 (PMC12423320; doi:10.1038/s44321-025-00285-5)
Supplement: Supplementary file 5 — Source data Fig. 3 [file 44321_2025_285_MOESM5_ESM.zip › Fig 3/3A/read me 3A.docx]

EPIC 850k Methylation data (idat and sdf files) available at:

Gene Expression Omnibus (GEO) GSE300902 (<https://www.ncbi.nlm.nih.gov/geo/query/acc.cgi?acc=GSE300902>).

Data sheet with sample names, heteropasmy levels, sentrix ID and position is available in excel data sheet.
