# Supplementary material for: Rewriting nuclear epigenetic scripts in mitochondrial diseases as a strategy for heteroplasmy control
Source: EMBO Mol Med. 2025 Aug 11;17(9):2354–83. doi: 10.1038/s44321-025-00285-5 (PMC12423320; doi:10.1038/s44321-025-00285-5)
Supplement: Supplementary file 5 — Source data Fig. 3 [file 44321_2025_285_MOESM5_ESM.zip › Fig 3/3B/read me 3B.docx]

Beta values used for fig 3B are available in dataset EV1 1 (beta values sheet).
