# Supplementary material for: Rewriting nuclear epigenetic scripts in mitochondrial diseases as a strategy for heteroplasmy control
Source: EMBO Mol Med. 2025 Aug 11;17(9):2354–83. doi: 10.1038/s44321-025-00285-5 (PMC12423320; doi:10.1038/s44321-025-00285-5)
Supplement: Supplementary file 7 — Source data Fig. 5 [file 44321_2025_285_MOESM7_ESM.zip › Fig 5/5A and 5B/read me 5A and 5B.docx]

5A. Data available in dataset EV1, metascape sheet.

5B. Data available in dataset EV1, ESR1 sheet.
