# Supplementary material for: Rewriting nuclear epigenetic scripts in mitochondrial diseases as a strategy for heteroplasmy control
Source: EMBO Mol Med. 2025 Aug 11;17(9):2354–83. doi: 10.1038/s44321-025-00285-5 (PMC12423320; doi:10.1038/s44321-025-00285-5)
Supplement: Supplementary file 9 — Source data Fig. 7 [file 44321_2025_285_MOESM9_ESM.zip › Fig 7/7B/readme 7B.docx]

Here are the fcs files for the flow cytometry image presented in fig 7B.

Autofluo= autofluorescence

FITC= cells labelled with Green CMFDA

LvH0= mixture of 13L labelled with green and unlabeled 13H treated with vehicle (DMSO)

LvHA= mixture of 13L labelled with green and unlabeled 13H treated with 5-azacytidine.

LvHD= mixture of 13L labelled with green and unlabeled 13H treated with decitabine.
