# Supplementary material for: Rewriting nuclear epigenetic scripts in mitochondrial diseases as a strategy for heteroplasmy control
Source: EMBO Mol Med. 2025 Aug 11;17(9):2354–83. doi: 10.1038/s44321-025-00285-5 (PMC12423320; doi:10.1038/s44321-025-00285-5)
Supplement: Supplementary file 10 — Source data Fig. 8 [file 44321_2025_285_MOESM10_ESM.zip › Fig 8/8C/read me 8C.docx]

Data for 8C is available in dataset EV2 and Tabix file, available at: https://www.mediafire.com/file/q9xjh6it3t0cu7s/Tabix.tsv.bgz/file
