# Supplementary material for: Rewriting nuclear epigenetic scripts in mitochondrial diseases as a strategy for heteroplasmy control
Source: EMBO Mol Med. 2025 Aug 11;17(9):2354–83. doi: 10.1038/s44321-025-00285-5 (PMC12423320; doi:10.1038/s44321-025-00285-5)
Supplement: Supplementary file 11 — Expanded View Figures [file 44321_2025_285_MOESM11_ESM.pdf]

Expanded View Figures

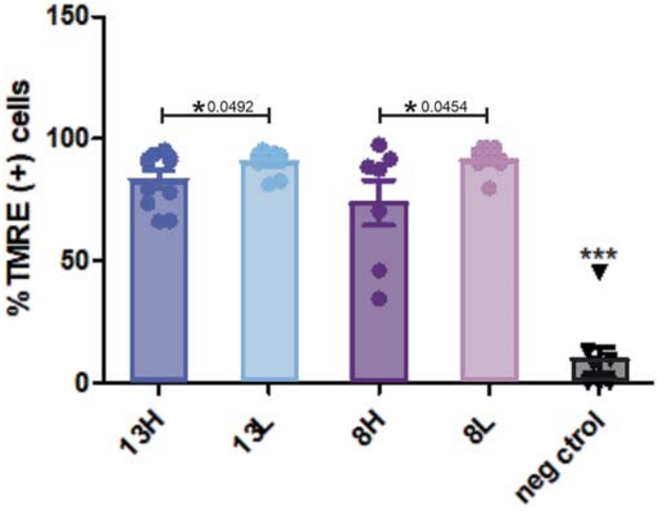

Figure EV1. Differences in mitochondrial membrane potential between high and low heteroplasmy cybrids.

Mitochondrial membrane potential measured with TMRE through Flow cytometry. Using a threshold based on autofluorescence in the TMRE-PE histogram, cells were designated as TMRE (+) or (−) for quantification. One-tailed unpaired Student´s *t* tests. N: 13H = 11, 13 L = 9, 8H = 7, 8 L = 7 independent experiments. *$P < 0.05$. Negative control CCCP. ***$P < 0.001$. Negative control significance is shown compared to the condition with the less difference (8H). Bars represent mean +/− SEM.

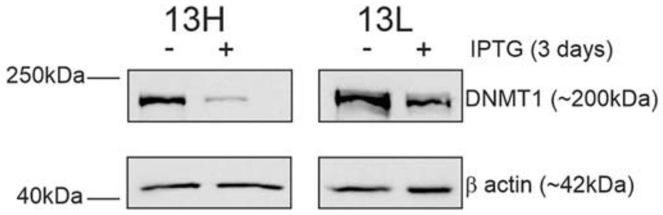

Figure EV2. DNMT1 knockdown control in ´13´ cybrids.

DNMT1 expression through Western Blot in the stable cell line of m.13513 G > A low (13 L) and high-heteroplasmy (13H) cybrids with the mammalian pLV[shRNA]-LacI: T2A:Puro-U6/2xLacO > hDNMT1[shRNA#1]. 3-day treatment with IPTG: isopropyl-galactosidase was installed to decrease DNMT1 expression.

 © The Author(s)

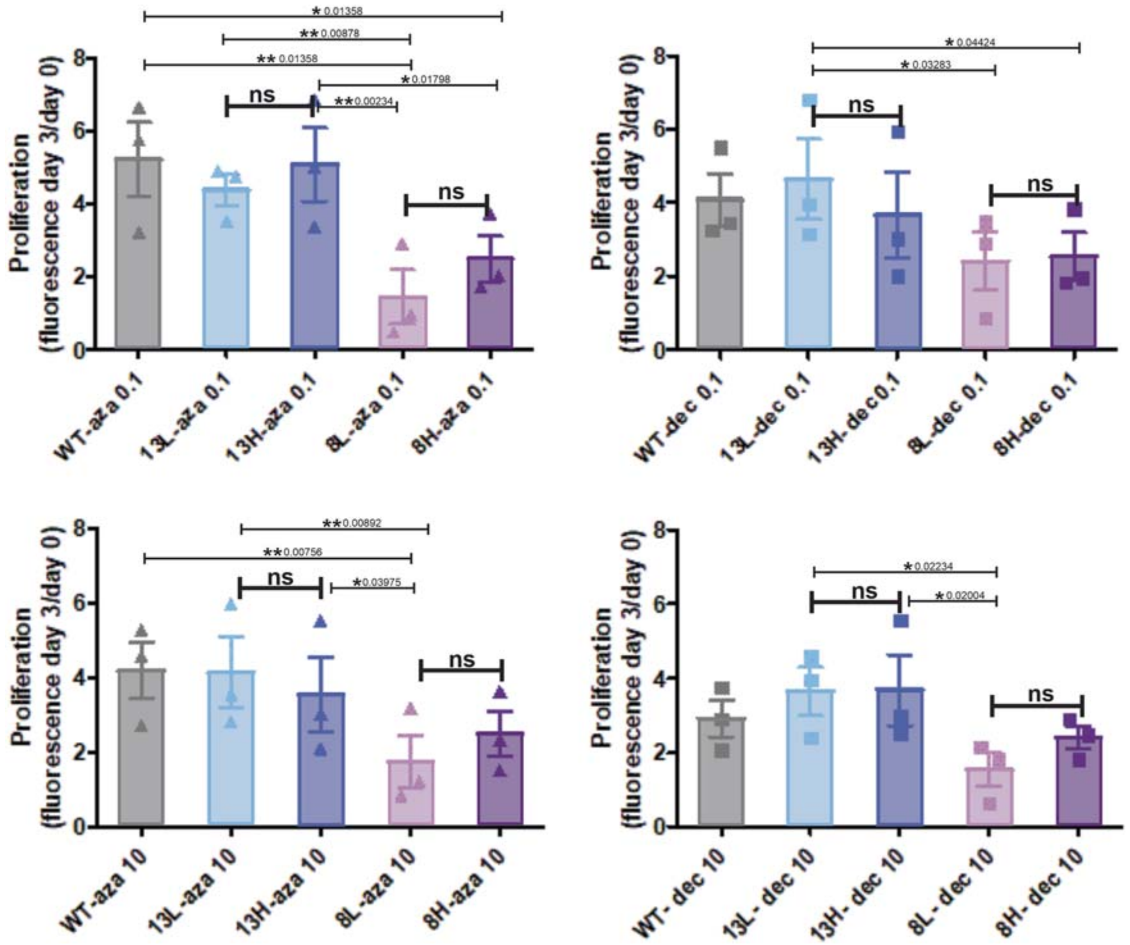

Figure EV3. DNA methylation inhibitors reduce differences in proliferation rates of high-low-heteroplasmy cybrids.

Differential proliferation of cells treated with 5-azacytidine (aza) and decitabine (dec) 0.1 µM (upper panels) or 10 µM (lower panels) for 3 days. Two-way ANOVA with fixed categorical variables, followed by Tukey's Honest Significant Difference test for post hoc comparisons. Residual normality was assessed using the Shapiro–Wilk test, implemented with the tidyr and nortest packages in R. All conditions passed the normality test. Comparisons between high and low heteroplasmies are stood out to compare with Fig. 4A. ns= not significant. $^{**}P < 0.01$, $^{*}P < 0.05$. $N = 3$ independent experiments, with technical duplicates. Bars represent mean +/− SEM.

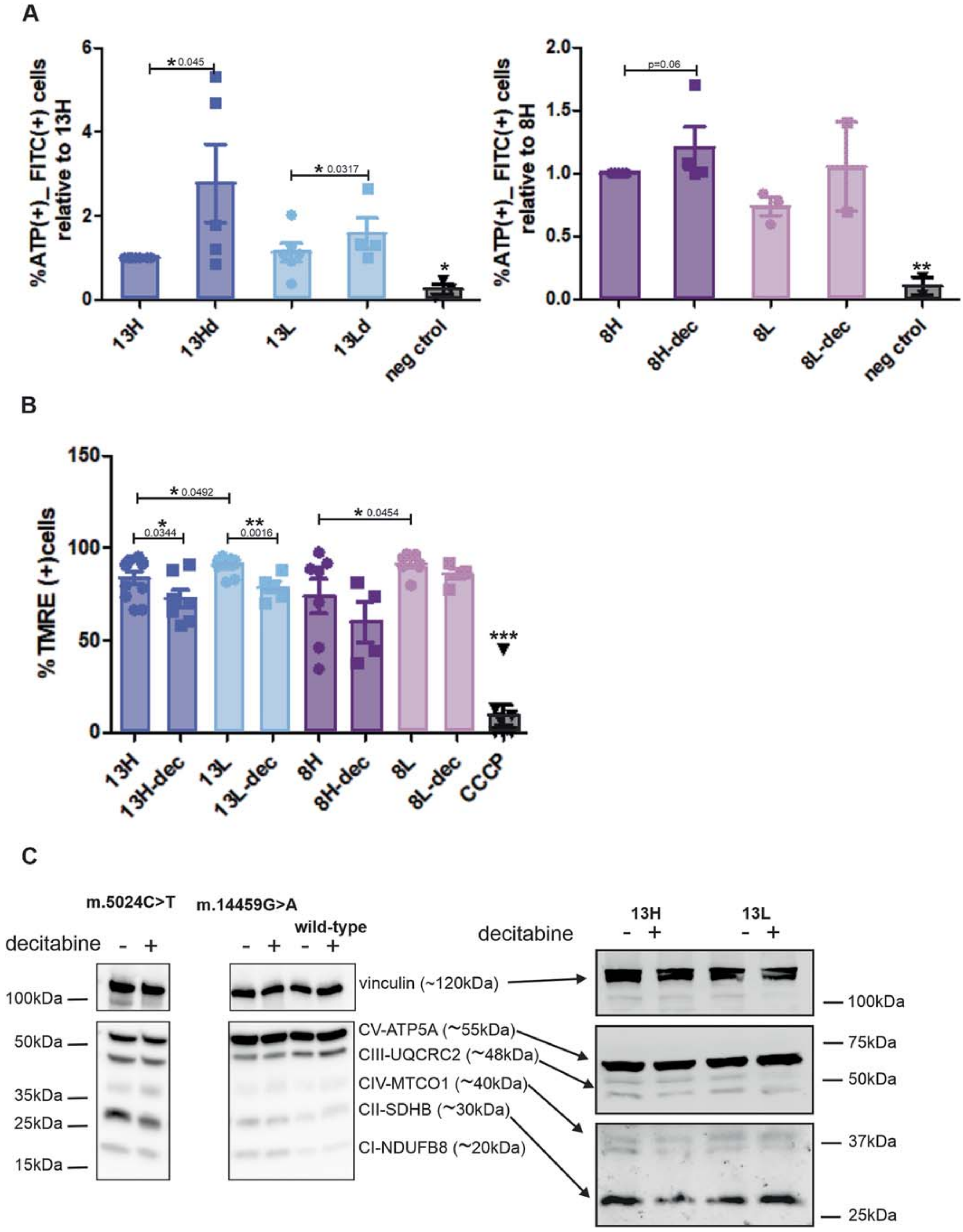
A
%ATP(+)_FITC(+) cells relative to 13H
*0.045
*0.0317
*
13H
13Hd
13L
13Ld
neg ctrol
%ATP(+)_FITC(+) cells relative to 8H
p=0.06
**
8H
8H-dec
8L
8L-dec
neg ctrol
B
% TMRE (+)cells
*0.0492
*0.0344
**0.0016
*0.0454
***
13H
13H-dec
13L
13L-dec
8H
8H-dec
8L
8L-dec
CCCP
C
m.5024C>T
m.14459G>A
wild-type
decitabine
13H
13L
vinculin (~120kDa)
CV-ATP5A (~55kDa)
CIII-UQCRC2 (~48kDa)
CIV-MTCO1 (~40kDa)
CII-SDHB (~30kDa)
CI-NDUFB8 (~20kDa)
100kDa
50kDa
35kDa
25kDa
15kDa
75kDa
37kDa

© The Author(s)

Figure EV4. DNA methylation inhibitor decitabine modifies mitochondrial function without altering the expression of mitochondrial complex proteins.

(**A**) Intracellular ATP content measured with Quinacrine through flow cytometry in cybrids treated 3 days with 1 µM. A threshold based on autofluorescence was set to divide cell populations as ATP (+) or (−) = FITC (+) or (−). The graph was outlined using values relative to the high-heteroplasmy sample (13H or 8H). *$P < 0.05$. One-tailed Wilcoxon matched-pairs signed rank test between unnormalized values of intra-experimental conditions. For 13 cybrids 3–7 independent experiments, for 8 cybrids 2–5. ATP content is shown significantly increased after treatment with decitabine in the 13 cybrids. A tendency to the same is shown in the 8. A control for ATP reduction was performed using CCCP+ Oligomycin, its´ significant fluorescent reduction is shown comparing to the sample with the lowest mean FITC (+) (13H and 8 L respectively), *$P < 0.05$, **$P < 0.01$. (**B**) Mitochondrial membrane potential measured with TMRE through Flow cytometry. Using a threshold based on autofluorescence in the TMRE-PE histogram, cells were designated as TMRE (+) or (−) for quantification. $N = 4$–11 independent experiments. One-tailed unpaired Student´s *t* tests. *$P < 0.05$, **$P < 0.01$. Negative control CCCP. ***$P < 0.001$. Negative control significance is shown compared to the condition with the less difference (8H). Bars represent mean +/− SEM. (**C**) Western blot to study the expression of mitochondrial complexes in different cells with and without decitabine treatment 1-day 1 µM. Left panel shows a gradient 4-20% SDS–polyacrylamide gel, right panel 12% SDS–polyacrylamide gel. In the 12% gel, CI-NDUFB8 was not visible. m.5024 C > T ~ 50% heteroplasmy MEFs, m.14459 G > A fibroblasts~95% heteroplasmy. 13H= m.13513 G > A high-heteroplasmy~80%, 13 L m.13513 G > A low-heteroplasmy~20%.

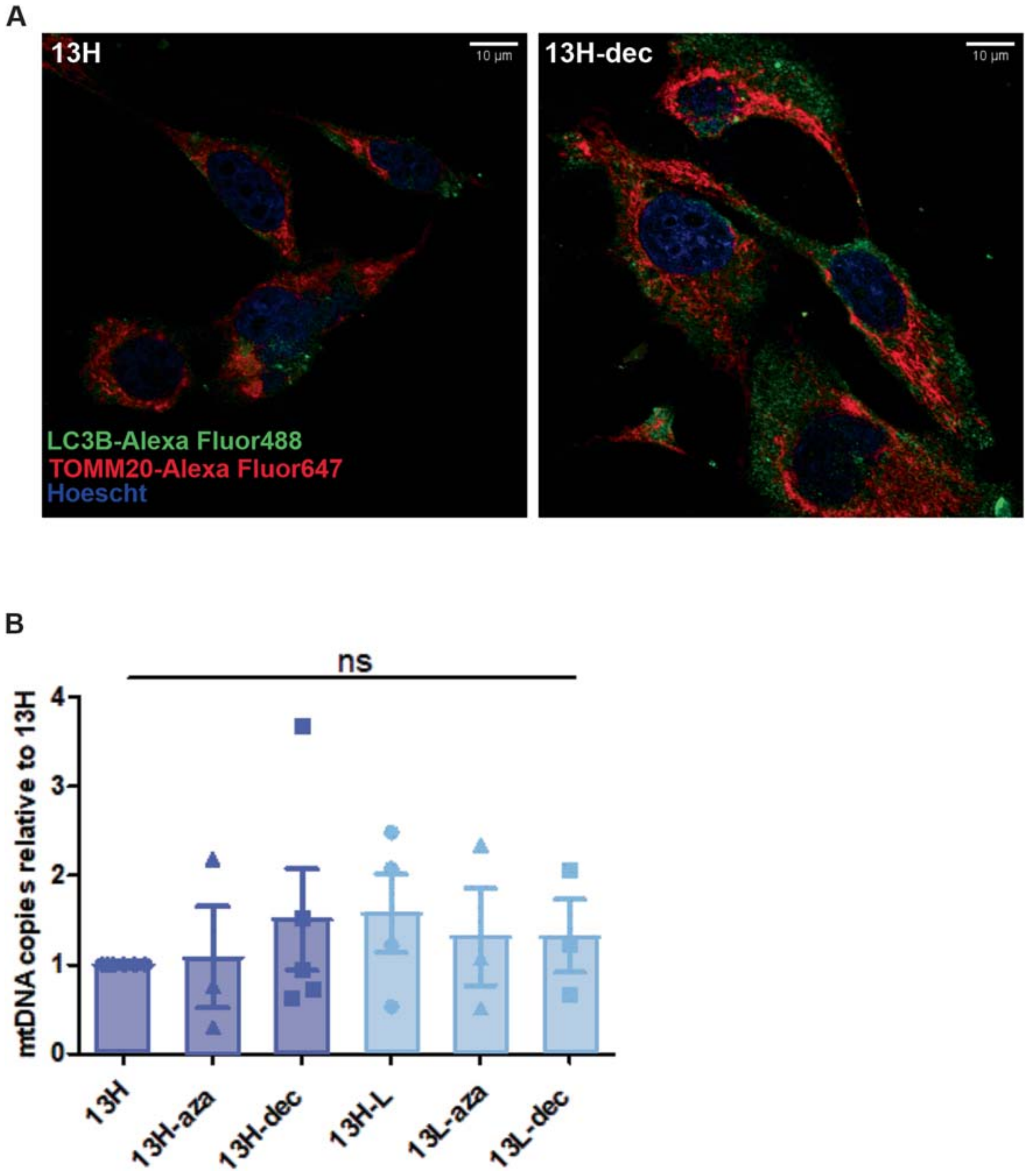

Figure EV5. DNA methylation modulators do not impact mitophagy nor mtDNA content.

(**A**) Confocal Immunofluorescence Microscopy. Confocal laser micrographs depict indirect immunofluorescence staining of LC3B (labeled with Alexa Fluor 488 in green) and TOMM20 (labeled with Alexa Fluor 647 in red) within m.13513 high-heteroplasmy cybrids (13H) treated with decitabine 1 µM—3 days (right panel) or DMSO (left panel). The cells´ nuclei were stained with Hoescht (blue). Colocalization of the two proteins is not evidenced. (**B**) mtDNA content. mtDNA content was measured with SYBR green-based RT-qPCR relativizing the m.13513 ´all´ amplicon to the nuclear gene B2M, quantified using ΔCT method= $2^{-(allCT-B2MCT)}$. Kruskal–Wallis test, Dunn's Multiple Comparison Test. $N = 3$–5 experiments. Bars represent mean +/− SEM.

 © The Author(s)

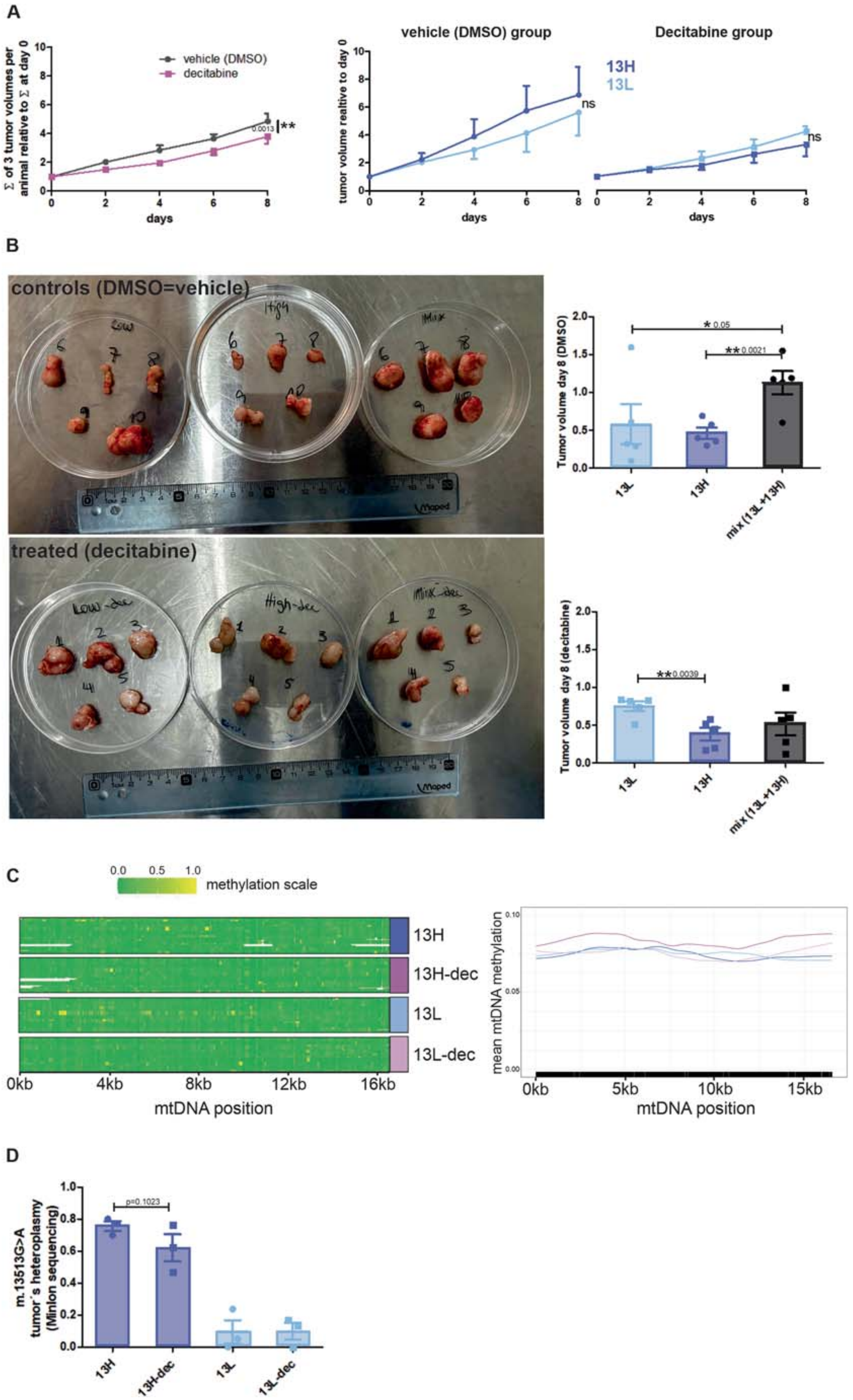
A
vehicle (DMSO)
decitabine
Σ of 3 tumor volumes per animal relative to Σ at day 0
days
0.0013
**
vehicle (DMSO) group
Decitabine group
13H
13L
ns
tumor volume realtive to day 0
B
controls (DMSO=vehicle)
treated (decitabine)
Tumor volume day 8 (DMSO)
Tumor volume day 8 (decitabine)
* 0.05
** 0.0021
** 0.0039
13L
13H
mix (13L+13H)
C
methylation scale
13H
13H-dec
13L
13L-dec
mtDNA position
mean mtDNA methylation
D
p=0.1023
m.13513G>A tumor´s heteroplasmy (Minion sequencing)

Figure EV6. Further characterization of xenograft experiments regarding tumor growth, tumor volume, mtDNA methylation and heteroplasmy.

(**A**) Left panel: overall tumor growth in mice in the control (DMSO) and treated (Decitabine) group. The volumes of the 3 tumors per animal were added up every measurement day and that sum was relativized to the sum of tumor volume of day 0 per animal. $^{**}P < 0.01$, two-way ANOVA test. Right panel: comparison of the high and low-heteroplasmy tumor growth within the control and treated group. (**B**) Final tumor size assessment at day 8. It is worth clarifying that tumor sizes started out uneven in order to have all 10 mice under treatment at the same time. Therefore, final tumor volumes *do not* represent their growth rate. Anyhow, we can stand out that the mixture of 13H + 13L grew to higher volumes (many ulcerating the skin -3 out of 5 mice-) when left untreated, upper panel. Also, within the treated mice (lower panel), the low-heteroplasmy tumors were less affected by decitabine than the high-heteroplasmy ones, $^{*}P < 0.05$. One-tailed, unpaired Student´s *t* test. (**C**) mtDNA methylation assessment using Nanopore sequencing technology. Coverage of the whole mitochondrial genome was satisfactory and showed low methylation values that were similar in all four conditions. No significant differences were observed, however, paradoxically the 13H-dec condition showed higher trend in methylation values than its untreated counterpart. $N = 3$ tumors from each condition: 13H, 13H-dec, 13L, 13L.dec. (**D**) Heteroplasmy assessment through Nanopore sequencing. Base call counts at position m.13513 are available in Dataset EV2. Heteroplasmy was calculated as ´A´ counts/(´A´counts + ´G´ counts). $N = 3$ tumors from each condition: 13H, 13H-dec, 13L, 13L.dec. Bars represent mean +/− SEM.

 © The Author(s)
